# Effect of the Mode of Application of Cryopreserved Human Amniotic Membrane on Adhesion Formation after Abdomino-Pelvic Surgery in a Mouse Model

**DOI:** 10.3389/fmed.2016.00010

**Published:** 2016-03-29

**Authors:** Joseph Nassif, Sehrish A. Abbasi, Mohamad Karim Kechli, Suzan S. Boutary, Labib Ghulmiyyah, Ibrahim Khalifeh, Hussein Abou Ghaddara, Anwar H. Nassar

**Affiliations:** ^1^Department of Obstetrics and Gynecology, American University of Beirut Medical Center, Beirut, Lebanon; ^2^Department of Anatomy, Cell Biology and Physiology, American University of Beirut Medical Center, Beirut, Lebanon; ^3^Department of Pathology, American University of Beirut Medical Center, Beirut, Lebanon; ^4^Department of Internal Medicine, American University of Beirut Medical Center, Beirut, Lebanon

**Keywords:** human amniotic membrane, abdomino-pelvic surgery, adhesion, mouse model, lactated ringer

## Abstract

Adhesions after abdomino-pelvic surgery are a cause of morbidity and reoperations. The use of human amniotic membrane (HAM) for adhesion prevention has given controversial results. The mode of administration of the amniotic membrane has not been well studied. This study assessed the efficacy of two modes of application of cryopreserved HAM, patch or fragmented in Lactated Ringer (LR) solution, for the prevention of pelvic adhesion formation postabdomino-pelvic surgery in a mice model. After a midline laparotomy incision, a small cautery lesion was done on each side of the abdominal wall peritoneum in mice. In Group A (control; *n* = 42), the abdomen was closed directly, Group B (*n* = 42) received 2.5 ml of LR prior to closure. In Groups C (*n* = 42) and D (*n* = 42), a 2 cm × 2 cm patch of HAM and another one fragmented and dispersed in 2.5 ml of LR were applied prior to closure, respectively. Two weeks later, a laparotomy was performed, and gross and pathological evaluation of adhesions, fibrosis, angiogenesis, and inflammation were conducted. Group D exhibited a significantly lower rate of gross adhesion formation. Fibrosis was significantly lowest in Group C as compared to the control. Group B had the lowest vascular formation in the adhesions. The use of HAM fragmented in LR solution is associated with a significantly lower incidence of postoperative adhesions in mice when compared to LR alone, HAM patch, or control. The mechanism of action of this reduction needs to be elucidated by future studies.

## Introduction

Adhesions are considered a major postoperative complication of abdominal and pelvic surgeries. They lead to significant clinical morbidities ranging from infertility, small bowel obstruction, pelvic pain, and future surgical complications (1, 2). Several agents have been proposed to decrease their occurrence, including antibiotics, non-steroidal anti-inflammatory drugs (NSAIDs), corticosteroids, fibrinolytics, reactive oxygen species (ROS) scavengers, calcium channel blockers, and GnRH analogs (3). Most of these agents have only been tested in animals, with some showing positive results; however, data from human trials are very limited. Several synthetic agents, which create a barrier between injured tissues throughout the peritoneal healing timeframe, have been proposed and used as adhesion prophylaxis agents. They can be site-specific (mechanical) or broad-coverage (gel or fluid) agents (2–4). Although some of these agents effectively reduce postsurgical adhesions, their use has many drawbacks, including difficulty in applying such agents in open or laparoscopic surgeries, decrease in their efficacy in the presence of bleeding, need for special and complex equipment for their application, and the high cost of most of those agents (1–3). Natural membranes such as omentum or peritoneal membranes have also been used but owing to their immunogenicity, they cannot be an ideal heterograft (5).

Human amniotic membrane (HAM) is a collagen avascular matrix with a basement membrane and a monolayer of epithelial cells and harbors macrophages and myofibroblasts. Many characteristics render it ideal to be used in adhesion prophylaxis since it is almost always available, easy to harvest, prepare, and sterilize, and it can be stored easily for several months at −80°C (6). HAM acts in two ways: suppression of adhesion formation by suppressing stromal inflammation, angiogenesis, fibrosis, and scarring (7) and enhancing wound healing by promoting epithelialization of the injured sites. HAM expresses HLA-A, HLA-B, HLA-C, HLA-G antigens, but not the HLA-DR antigen or B2-microglobulin (5, 6, 8, 9), and has anti-inflammatory and anti-microbial activities (5, 7). In the past few years, HAM has been used as biological membranes in the treatment of burns, lesions, and ulcers (6, 9); surgical repair of artificial vagina; and omphalocele repair (6, 10). HAM is most widely used in ophthalmic surgeries and in the treatment of various diseases of the external eye (11).

Various studies have assessed the utilization of HAM in adhesion prophylaxis alone or in combination with other pharmaceutical agents (4, 5, 12). Most of these studies have utilized the HAM as a patch (13, 14). HAM has also been tested with agents such as heparin, Seprafilm (15), and several other substances to look for an increased anti-adhesion action (16–18). In addition, it was compared to hyaluronate/carboxymethylcellulose membrane (19). However, to the best of our knowledge, HAM has not been compared to the Lactated Ringer (LR) solution and has not been studied in the fragmented form in LR solution. The aim of our study is to assess the efficacy of the mode of application of the HAM, patch or fragmented in LR solution, in preventing postoperative adhesion formation in a mouse model compared with LR alone.

## Materials and Methods

### Ethics Statement

The American University of Beirut’s Institutional Review Board and the Institutional Animal Care and Use Committee approved the study. All the research was conducted in compliance with The Code of Ethics of the World Medical Association (Declaration of Helsinki) for experiments involving humans, and EU Directive (2010/63/EU) for animal experiments. All prospective patients were informed about the study in detail, and an informed consent was signed prior to the procedure by those willing to donate the amniotic membrane.

### Sample Size Calculation

With a hypothesized effect size of 50% and under the assumptions of a type-I error (two sided) of 5% and a power of 90%, 42 mice per arm for the control and different therapeutic groups were estimated to be needed. Using a computer generated list, mice were randomized into four groups (A: control, B: LR, C: HAM patch, and D: fragmented HAM in LR) of 42 mice each.

### Patient Recruitment

Human amniotic membranes were collected from five consecutive patients undergoing an elective cesarean delivery. The patients consented to donate the placentae and be tested for HCV, HIV1 and 2, and HbsAg. In addition to testing negative for these tests, all patients had no evidence of any genital infectious diseases, fever (>38°C), premature rupture of the membranes, and meconium-stained amniotic fluid.

### Collection and Storage of the Human Amniotic Membranes

The placentae were collected after each elective cesarean delivery and were transported in sterile bags on ice to the biomolecular lab, where they were bluntly dissected from the chorion under the laminar flow hood. They were washed with balanced salt solution containing penicillin, streptomycin, neomycin, and amphotericin B, placed in tissue culture and glycerol at a ratio of 1:1, and stored at −80°C.

### Processing of the Human Amniotic Membranes

Human amniotic membrane was processed in two different ways for the procedures. For the group receiving the HAM patch, HAM was cut into a 2 cm × 2 cm patch, which was then suspended in phosphate buffer saline (PBS) and directly sent to the operating room in the animal care facility in a sterile bag on ice. For the fragmented HAM patch in LR group, a 2 cm × 2 cm HAM patch was suspended in PBS, centrifuged, and the resultant fragment HAM mixture was put in 2.5 ml of LR, and sent to the operating room in the animal care facility in a sterile bag on ice.

### Surgical Procedures

A total of 168 female BALB/c mice weighing 20–25 g were used in this study. The animals were kept in the animal house of the American University of Beirut according to the international principles of animal care. The mice were ordered in batches of 20 mice each, in periods ranging from 2 to 3 weeks.

Mice were chosen as the study model due to lack of a xenogeneic reaction after HAM transplantation, as seen in the Zhang et al. study (20). This study investigated immunological and histological reactions in mice after xenotransplantation of either fresh HAM or preserved HAM, and compared the results between the two groups. The result showed that HAM, fresh or preserved, has ideal immunocompatibility and histocompatibility as a heterologous biomaterial.

All procedures were performed by the same surgeon, Mohamad Karim Kechli, experienced in surgery on mice, and relevant images were captured using an Olympus SC30 camera. Abdominal and pelvic areas were shaved 1 day prior to the surgical procedure. Cefazolin (400 mg/kg) was given 30 min prior to the incision for infection prophylaxis. All mice received induction of anesthesia using a combination of intramuscular ketamine 90–100 mg/kg (Ketalar, Parke-Davis, Morris Plains, NJ, USA) and xylazine 10 mg/kg (Gemini, Rugby Laboratory, Rockville Center, NY, USA). Mice were draped in a sterile fashion and scrubbed using iodine povidone solution. A midline incision of 3 cm was then performed. Two experimental injuries, measuring 0.5 cm each, were performed using electrocautery at 20 W (Valleylab Generator) applied for 1 s on each side of the internal aspect of the abdominal wall.

**Figure 1:**
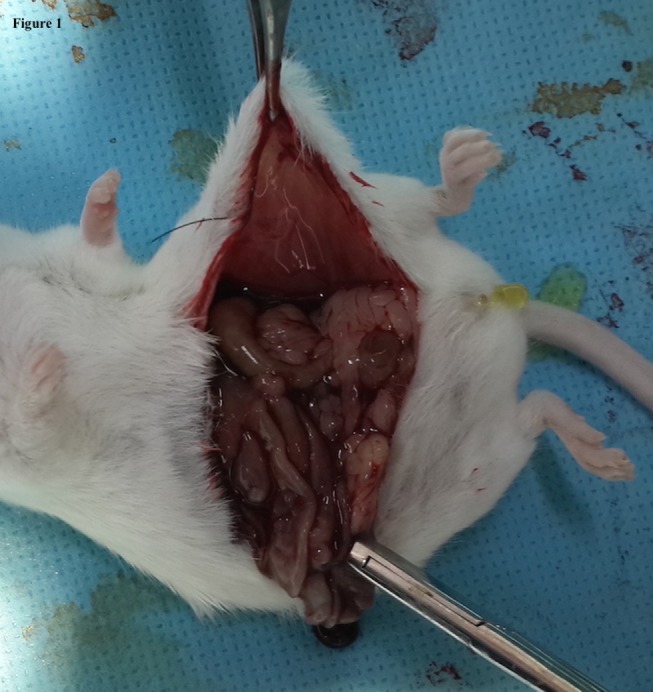
**Aspect of a 2 cm × 2 cm HAM patch left over the cauterized area (Group C) prior to closure on the day of surgery**.

In the control (Group A), after a midline laparotomy incision of 3 cm length, a small cautery lesion of 0.5 mm was done on each side of the peritoneum of the abdominal wall, which was then closed. Group B underwent the same procedure, but 2.5 ml of LR were put in the abdominal cavity prior to closure. In Group C, a patch of 2 cm × 2 cm of HAM was left over the cauterized areas prior to closure (Figure 1). Group D received the 2 cm × 2 cm patch of HAM fragmented and dispersed in a 2.5 ml of LR prior to closure. The abdomen was closed using 5-0 nylon interrupted sutures, and the wound was covered with Mebo^®^ ointment (Julphar Gulf Pharmaceutical, Amman, Jordan).

### Pathological Analysis

On postoperative day 14, the mice received a fatal dose of furan gas, and an autopsy was performed to grossly assess the extent of the adhesions. The adhesions were grossly assessed by the surgeon according to the modified Nair’s macroscopic adhesion classification: grade 0, no adhesions; grade 1, a single adhesive band between organs or organs and abdominal wall; grade 2, two adhesive bands between organs or organs and abdominal wall; grade 3, more than two adhesive bands between organs and abdominal wall, or adhesion to intestinal loops without adhesions to abdominal wall; and grade 4, thick and complex adhesive band between organs or organ and abdominal wall or direct adhesion of viscera to abdominal wall. The results are shown in Table 1.

**Table 1:** **Gross adhesion results**.

	Gross adhesion percentage (%) (*n*)			
Grade	Group A	Group B	Group C	Group D
0	0 (0)	7.7 (3)	5.1 (2)	71 (27)
1 and 2	37.5 (15)	53.8 (21)	46.1 (18)	15.7 (6)
3 and 4	62.5 (25)	38.5 (15)	48.8 (19)	13.3 (5)
Total surviving mice	40	39	39	38
Total sampled mice	40	36	37	11

If adhesions were present, an adhesion sample was sent for pathology studies. One pathologist (IK), blinded to the group of specimen, read all the Hematoxylin & Eosin (H&E)-stained histological slides using an Olympus BX40 microscope at a magnitude of 100×. The sample slides were fixated in formalin and assessed for fibrosis, angiogenesis, and inflammation levels, according to the scores shown in Table 2. The inflammation was graded as: no inflammation, scattered infiltrates, or continuous infiltrates; the fibrosis as: no fibrosis, patchy scattered bands, or dense bands; and the angiogenesis as: no vascular proliferation, patchy vascular proliferation, and dense vascular proliferation.

**Table 2:** **Adhesion sample pathological grading criteria**.

Score	Histological features		
	Inflammation	Fibrosis	Angiogenesis
0	No inflammation	No fibrosis	No vascular proliferation
1	Scattered infiltrates	Patch scattered bands	Patchy vascular proliferation
2	Continuous infiltrates	Dense bands	Dense vascular proliferation

Data were entered to the PASW (SPSS version 18.0). A *p*-value <0.05 was considered statistically significant. Statistical analysis was done using ANOVA (analysis of variance) for comparing multiple variables. For *post hoc* analysis, two-sample *t*-test was used to determine the difference between the groups.

## Results

### Mortality

Two mice died in Group A, whereas Groups B, C, and D had 3, 3, and 4 deaths, respectively. The overall *p*-value for mortality among the groups was 0.87, which shows that there is no significant difference among the groups in terms of mortality, Table 3. All mice died within 12 h of the procedure and the only plausible cause was anesthetic complication. There were no infections, no seromas, and no dehiscence.

**Table 3:** **Association between the four groups and the outcomes**.

Variables	Score	Group A	Group B	Group C	Group D	*p* value	*p* value Group B versus Group A	*p* value Group C versus Group A	*p* value Group D versus Group A	*p* value Group D versus Group C
Total sample		*n* = 42	*n* = 42	*n* = 42	*n* = 42					
Total mice sampled for adhesions^a^		*n* = 40	*n* = 36	*n* = 37	*n* = 11					
Inflammation	0 No inflammation	16 (40.0%)	17 (47.2%)	7 (18.9%)	4 (36.3%)	0.11	0.81	0.06	0.32	0.40
	1 Scattered infiltrates	18 (45.0%)	14 (39.0%)	18 (48.7%)	4 (36.3%)					
	2 Continuous infiltrates	6 (15.0%)	5 (13.8%)	12 (32.4%)	3 (27.4%)					
Fibrosis	0 No fibrosis	14 (35.0%)	16 (44.5%)	7 (18.9%)	3 (27.4%)	0.03	0.68	0.04	0.10	0.55
	1 Patchy scattered bands	22 (55.0%)	17 (47.2%)	18 (48.7%)	5 (45.2%)					
	2 Dense bands	4 (10.0%)	3 (8.3%)	12 (32.4%)	3 (27.4%)					
Angiogenesis	0 No vascular proliferation	13 (32.5%)	29 (80.5%)	9 (24.4%)	4 (36.3%)	<0.0001	<0.0001	0.19	0.14	0.54
	1 Patchy vascular proliferation	22 (55.0%)	5 (13.8%)	17 (45.9%)	4 (36.3%)					
	2 Dense vascular proliferation	5 (12.5%)	2 (5.7%)	11 (29.7%)	3 (27.4%)					
Death	No	40 (95.2%)	39 (92.9%)	39 (92.9%)	38 (90.5%)	0.87	1.00	1.00	0.68	1.00
	Yes	2 (4.8%)	3 (7.1%)	3 (7.1%)	4 (9.5%)					
Gross inspection	(Mean ± SD)	2.7 (±1.0)	2.0 (±1.1)	2.2 (±1.2)	1.5 (±1.0)	<0.0001	0.004	0.03	<0.0001	0.008

*Group A: (control) the abdomen was closed directly; Group B: received 2.5 ml of Lactated Ringer prior to closure; Group C: a patch of 2 cm × 2 cm of HAM was left over the cauterized areas prior to closure; Group D: received the 2 cm × 2 cm patch of HAM fragmented and dispersed in a 2.5 ml of LR prior to closure*.

*^a^Sampled adhesions were studied for inflammation, fibrosis, and angiogenesis*.

### Gross Adhesions

For analysis purposes, the adhesion grades assessed using Nair’s macroscopic adhesion classification were grouped into three categories (0, 1 and 2, 3 and 4). In the control Group A, all mice developed gross adhesions, whereas Group D had the highest rate of no gross adhesions at 71%. In Group D, 15.7% of mice developed grades 1 and 2 adhesions, compared to 37.5% in Group A. Groups B and C had almost similar rates of grades 1 and 2 adhesions. In terms of grades 3 and 4 adhesions, the highest percentage of 62.5% was found in Group A and the lowest in Group D at 13.3%, Table 1.

**Figure 2:**
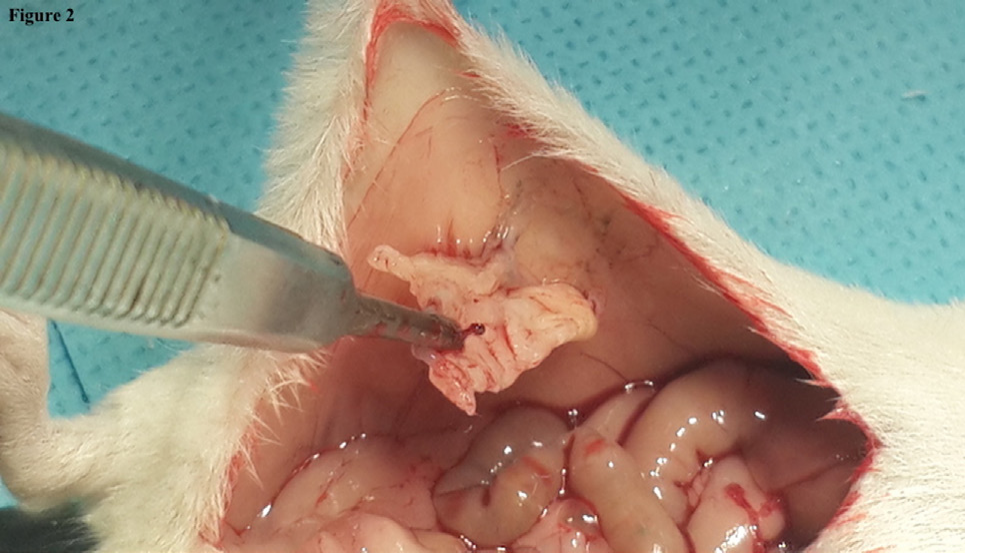
**Aspect of the amniotic membrane patch (Group C) on autopsy showing a score 2 adhesions**.

**Figure 3:**
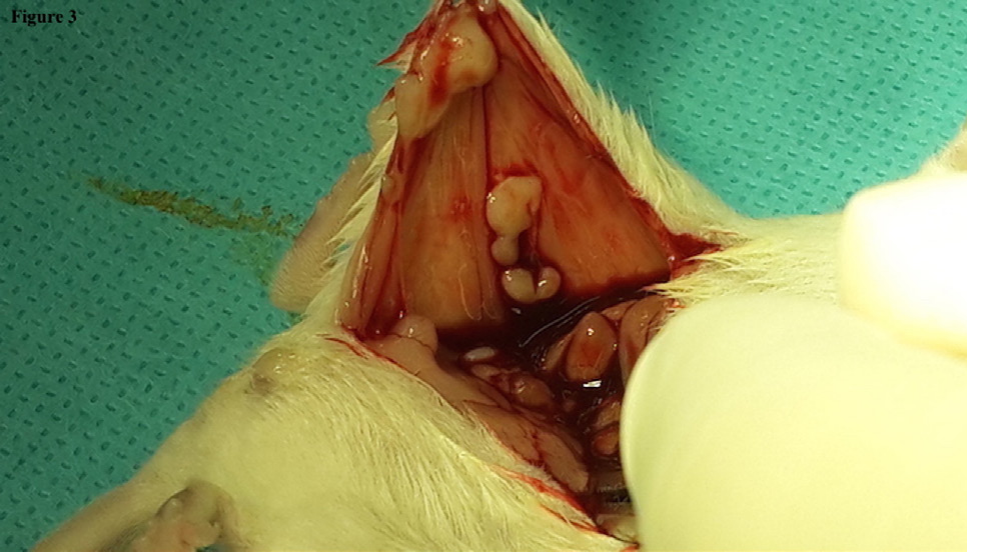
**Aspect of the fragmented amniotic membrane in Lactated Ringer (Group D) on autopsy showing a score 1 adhesions**.

The lowest mean for gross adhesions was found to be in Group D (1.5 ± 1.0 SD) followed by Groups B (2.0 ± 1.1 SD), C (2.2 ± 1.2 SD), and A (2.7 ± 1.0 SD). The overall *p* value of <0.0001 indicated a significant difference in gross adhesion formation between the groups. *Post hoc* analysis showed significant decrease in gross adhesion formation between the fragmented HAM (Group D) and the control Group A. Also, a comparison between Groups C and D (Figures 2 and 3) gave a *p*-value of 0.008 suggesting that there was a significant decrease in gross adhesion formation in Group D as compared to the HAM patch Group C, Table 3.

### Pathology Results

**Figure 4:**
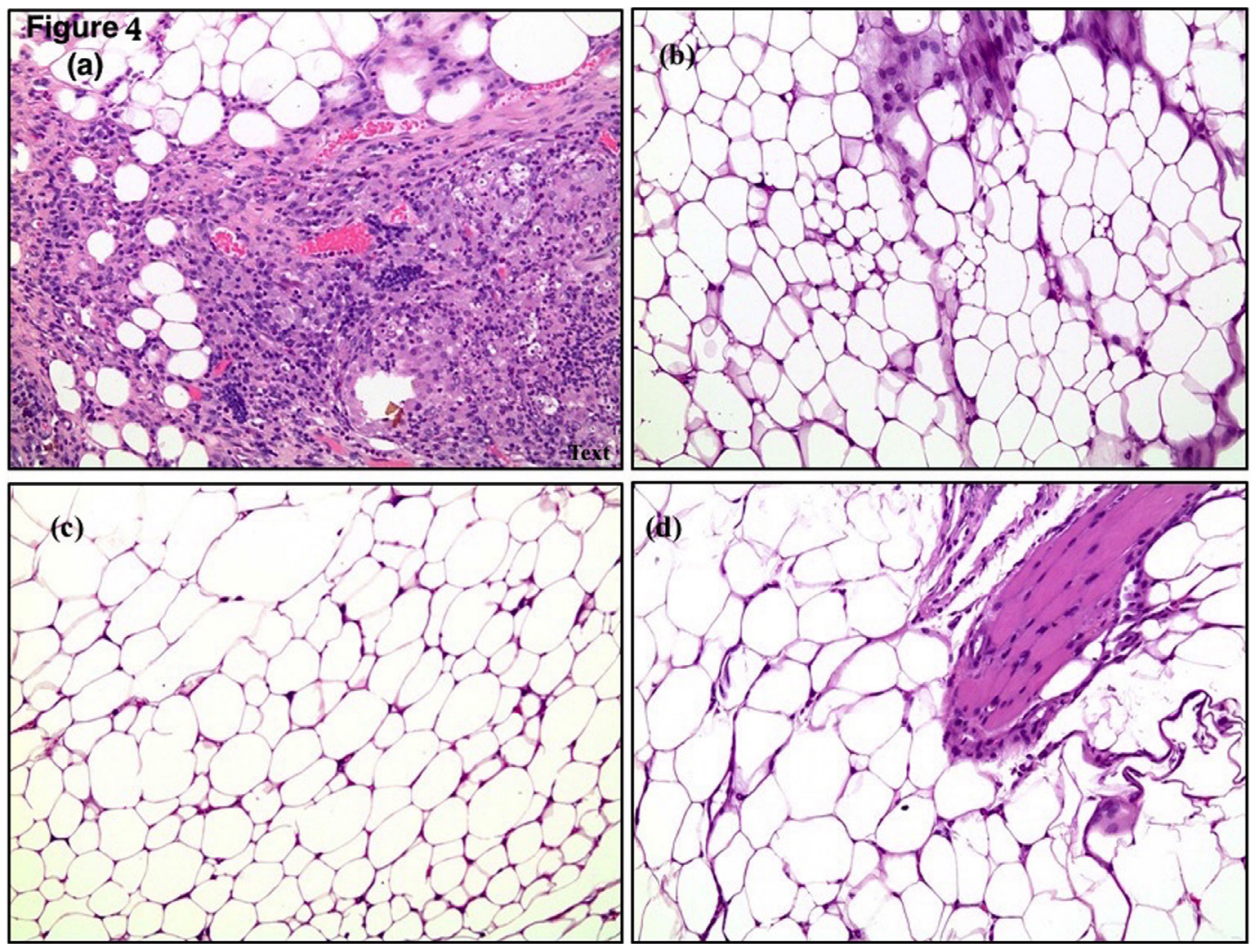
**Histological slides from fragmented HAM in LR mice showing (A) fibrosis, (B,C) inflammation, (D) neoangiogenesis**.

All mice in the control Group A developed adhesions and were all sampled. In total, 92.3, 94.9, and 29% of mice in Groups B, C, and D, respectively, developed adhesions and were sampled (Table 1). Sampled adhesions were studied for inflammation, fibrosis, and angiogenesis. The pathology results of the sampled adhesions, when present, are shown in Table 3 (Figure 4).

In terms of inflammation, the least adhesion infiltrates were found in Group B, where 47.2% of the mice did not develop infiltrates, whereas only 18.9% of Group C did not develop infiltrates. Group C had the highest rate of scattered and dense infiltrates at 48.7 and 32.4%, respectively. The overall *p*-value for inflammation in the groups was calculated to be 0.11(*p*-value >0.05); therefore, indicating that there is no actual significant difference amongst the four groups in terms of inflammation.

Group B had the least fibrosis among the groups where 44.5% of mice did not develop fibrosis, 47.2% had patchy scattered bands, and 8.3% had dense bands. Similar results were obtained for the patchy scattered bands in the four groups (45–55%), with the least patchy scattered bands found in the Group D. Groups A and B had similar rates of dense bands, 10 and 8.3%, respectively, and Groups C and D had 32.4 and 27.4% of dense bands, respectively. The overall *p*-value of 0.03 indicates that there is a significant difference among the groups in terms of fibrosis and that Group C has significantly lower fibrosis rates as compared to the control (*p*-value <0.05 = 0.04). Group B had the least fibrosis rates; however, the *p*-value of 0.68 (*p*-value >0.05) shows that there is no significant reduction in fibrosis in Group B as compared to the control.

For angiogenesis, overall Group B was associated with the least vascular formation in the adhesions. In total, 80.5% of mice in Group B did not develop angiogenesis in the adhesions, 13.8% had patchy vascular infiltration, and 5.7% had dense vascular infiltration. The overall *p*-value was calculated to be <0.0001 suggesting a significant difference among the groups in terms of angiogenesis. Group B had a *p*-value of <0.0001 when compared to Group A, showing statistical significance, whereas no other group had a statistically significant difference compared to the control.

## Discussion

In our study, Group D (fragmented HAM in LR) had the least rate of gross adhesion formation, where 71% of the mice did not develop adhesions. Fibrosis was significantly lowest in Group C as compared to the control. Lowest vascular formation in the adhesions was seen in Group B, where 80.5% of mice did not develop angiogenesis in adhesions.

The use of dried HAM in adhesion prevention has been tested in animal and human applications (21). Patches of HAM have been used with the maternal side facing the injury and the patches fixed by cautery or sutures at the lesion site. Controversial results were reported by these studies (22). It was reported that the anti-adherent properties of HAM are likely to lie in the amniotic epithelium or in the properties that derive not only from its stem cells but also from its mechanical property as a barrier (21). Petter-Puchner et al. showed a possibility of remaining pluripotent cell activity after cryopreservation (4). LR has been previously shown to decrease adhesion formation in rats (23), which prompted us to compare it to the other forms of administration of HAM. To our knowledge, this comparison was not performed before up till now.

In this study, 100% of the Group A (control) developed gross adhesions, making it an ideal model for adhesion study. The HAM patch was not fixated by stitches or cautery in Group C in order to avoid the bias of introducing a confounding factor to the adhesion formation. The adhesion formation rate in this group is comparable to the Group B (LR group). This can be explained by the inability of the non-fixated patch to play its role as a barrier as it might have slipped from its application site after ambulation.

In Group D (fragmented HAM in LR), there was a significantly lower gross adhesion formation rate compared to the other groups, where only 29% of mice developed adhesions compared to 100, 92.3, and 94.9% of mice in Groups A, B, and C, respectively. This might be due to the larger contact surface between the different elements of HAM and the lesion. We hypothesize that fragmentation of HAM releases cells and collagen from HAM, leading to faster stimulation of epithelialization than the adhesion fibrin bands. Ambulation ensures a better distribution of the HAM’s components when suspended in LR as compared to the HAM patch. The combination of HAM and LR seems to have a synergistic effect.

Histological testing showed a similar inflammatory response in all groups, which was reported to be present even with the use of amniotic membranes by Di Loreto et al. (21). This suggests that the mechanism of action of all the anti-adhesion agents used in the study is independent of the inflammatory reaction. Angiogenesis was found to be lowest in the Group B; this can be explained by a potential anti-angiogenesis effect of LR solution. The significantly lower rate of fibrosis associated with the use of HAM patch cannot be explained with the current design of the study. The lack of a group where fragmented HAM was applied without LR is a limitation of the present study as it would have led to a better understanding of the fragmented HAM’s properties alone.

The results obtained in this study have a significant relevance in human setting as they can help prevent postsurgical abdomino-pelvic adhesions. Since, caesarian deliveries are associated with a high rate of postsurgical adhesions, amniotic membranes obtained from a patient undergoing a cesarean surgery can be fragmented in LR and placed in her abdomen right after the uterine closure and prior to the abdominal closure. This would help prevent postoperative abdomino-pelvic adhesions and subsequent complications. Cryopreserved HAM can also be used in other gynecological and abdominal surgeries for the same benefit. This study opens the field of research about the use of fragmented HAM in cesarean deliveries, gynecological and abdominal surgeries, in an autologous or homologous fashion.

The use of HAM fragmented in LR is associated with a significantly lower incidence of postoperative adhesions in mice when compared to LR alone, HAM patch, and control. Further studies are needed to understand the mechanism of action of the fragmented HAM.

## Author Contributions

JN: conceived and designed the study, helped in discussion and data analysis, and edited the manuscript. SA: wrote the manuscript and helped in discussion and data analysis. MK: performed surgeries and helped in discussion and data analysis. SB: helped perform surgeries and in discussion and data analysis. IK: performed pathological studies. AN, HG, and LG: helped in discussion and data analysis and edited the manuscript. All authors read and approved the final version of the manuscript to be submitted.

## Conflict of Interest Statement

This research was conducted in the absence of any commercial or financial relationships that could be construed as a potential conflict of interest.
